# Reconstitution of dominant kombucha strains to enhance functional properties and product quality

**DOI:** 10.3389/fnut.2025.1687776

**Published:** 2025-12-03

**Authors:** Ting Liao, Li Li, Jing-Rong Yang, Shi-Ting Shao, Wei-Ming Zheng, Lai-Hoong Cheng, Li Fan

**Affiliations:** 1 College of Tea and Food Science, Wuyi University, Wuyishan, China; 2 Food Technology Division, School of Industrial Technology, Universiti Sains Malaysia, Minden, Pulau Pinang, Malaysia; 3 Strait Success College, Wuyi University, Wuyishan, China

**Keywords:** kombucha, dominant strains, synthetic consortia, fermentation, sensory quality

## Abstract

**Introduction:**

Previous studies have shown that yeasts, acetic acid bacteria, and lactic acid bacteria (LAB) dominate the fermentation of kombucha. However, the functional contributions of defined microbial combinations remain insufficiently characterized. This study aimed to isolate dominant kombucha microorganisms and construct synthetic consortia to evaluate their effects on fermentation performance and product quality.

**Methods:**

Six dominant strains—*Saccharomyces cerevisiae* (JQ-1), *Pichia manshurica* (JQ-2), *Acetobacter papayae* (CQF1), *Acetobacter tropicalis* (CQF2), *Lactobacillus plantarum* (RXA1), and *Lactobacillus fermentum* (RXA2)—were isolated and reconstituted into three synthetic consortia. Fermentation products were analyzed for phenolic content, antioxidant capacity, organic acids, free amino acids, caffeine, sensory attributes, and microbial safety.

**Results:**

All synthetic consortia significantly increased phenolic content, antioxidant activity, and organic acid accumulation, while reducing free amino acids and caffeine levels. Among them, Consortium B (JQ-1 + JQ-2 + CQF1 + CQF2) achieved the highest sensory acceptability due to its balanced acidity and flavor profile. Microbial safety assessments confirmed the absence of pathogenic microorganisms.

**Discussion:**

These results demonstrate that rationally designed microbial consortia can effectively improve the functional quality, sensory properties, and safety of kombucha. The study provides a scientific basis for developing standardized and optimized industrial fermentation strategies.

## 1 Introduction

Kombucha is a traditional fermented tea beverage produced by a symbiotic consortium of yeasts and bacteria in sugared tea. It is valued not only for its refreshing taste but also for its reported health benefits, including digestive regulation, anti-inflammatory, antimicrobial, and antidiabetic effects. These functions are largely attributed to bioactive metabolites such as organic acids, polyphenols, vitamins, and amino acids, which are generated during microbial fermentation (1, 2).

The kombucha microbiota is dominated by yeasts and acetic acid bacteria, with lactic acid bacteria (LAB) frequently present (3, 45). In China, production still relies mainly on natural static fermentation, during which microorganisms metabolize sucrose and tea-derived nutrients, forming a cellulose-based biofilm within about 21 days (4). However, the use of whole kombucha pellicle as inoculum leads to large variations in microbial composition, resulting in inconsistent fermentation and product quality.

In recent years, advances in culture-dependent isolation methods and high-throughput sequencing have clarified the core microbiota and metabolic dynamics of kombucha (5, 6). However, relatively few studies have investigated the ecological interactions among dominant strains and their combined contributions to functional properties. Synthetic microbial consortia have been proposed as a promising approach to overcome the limitations of uncontrolled inoculation, offering advantages in stabilizing fermentation, regulating organic acid production, and enhancing health-related metabolites (7, 45). Similar strategies have been successfully applied in other fermented foods and beverages, such as sourdough, cheese, and wine, where rationally designed consortia improve consistency and product quality.

Despite progress in identifying the core microbiota of kombucha and understanding their metabolic roles, there remains a lack of systematic evaluation of how reconstituted microbial consortia influence both functional properties and product safety. To address this gap, the present study aimed to isolate dominant strains from traditional kombucha and reconstitute them into defined microbial consortia. We specifically sought to evaluate their fermentation performance, metabolite production (including organic acids, polyphenols, antioxidants, amino acids, and caffeine), sensory attributes, and microbial safety. Furthermore, these outcomes were compared to those obtained from traditional back-slopping fermentation. By clarifying microbial interactions and functional outputs, this work provides a theoretical foundation for the development of standardized and reproducible kombucha production strategies.

## 2 Materials and methods

### 2.1 Traditional kombucha liquid

Purchased from a Taobao store in China (Shandong Chi's Kombucha Stomach Treasure Vinegar Moth), the liquid was stored in a laboratory 4 °C refrigerator for use.

### 2.2 Separation and purification of yeast

Rationale for isolate selection: the number of isolates was restricted to two per microbial group to capture functional diversity while maintaining experimental feasibility. This strategy was informed by prior studies linking kombucha microbial diversity with metabolite dynamics. From multiple colonies initially obtained, only isolates that demonstrated consistent colony morphology, robust growth stability across repeated subculturing, and reproducible metabolic activity in preliminary tests were retained.

Kombucha broth was serially diluted (10^1^-10^3^) and plated on Bengal red agar (Peptone: 0.5%, Glucose: 1.0%, Potassium Dihydrogen Phosphate: 0.1%, Magnesium Sulfate (containing 7H_2_O): 0.05%, Agar: 2.0%, Bengal Red: 0.00333%, Chloramphenicol: 0.01%, Oxytetracycline: 0.005%). After incubation at 30 °C for 48 h, yeast-like colonies were purified on YPD media (Peptone: 2.0%, Glucose: 2.0%, Yeast Extract: 1.0%, pH: 6.5 ± 0.2 (25 °C), Oxytetracycline: 0.005%). Two isolates (JQ-1, JQ-2) were cultured in YPD broth (32 °C, 180 r/min, 20 h) and preserved in 80% glycerol at −80 °C. Morphological features were examined microscopically (40×).

### 2.3 Isolation and purification of acetic acid bacteria

Kombucha broth was serially diluted (10^1^−10^3^), and 50 μl of each dilution was spread on acetic acid bacteria isolation medium. After incubation at 32 ± 1 °C for 2 days, colonies with discoloration halos were purified by repeated subculture. Two isolates (CQF1, CQF2) were obtained. Gram staining was performed, and cell morphology was observed under an oil immersion microscope (SMART, Chongqing Aote Optical Instrument Co., Ltd., Chongqing, China (8).

### 2.4 Isolation and purification of lactic acid bacteria

Kombucha broth was serially diluted (10^1^-10^3^), and suspensions were spread on MRS agar plates. After incubation at 37 °C for 48 h, colonies with distinct morphology were purified and examined by Gram staining and microscopy. Two Gram-positive isolates were obtained (RXA-1, RXA-2). Cultures were preserved in 20% glycerol at −80 °C (46).

### 2.5 Molecular identification of microorganisms in kombucha

#### 2.5.1 Bacteria

Acetic acid and lactic acid isolates were cultured in MRS broth (5 ml, 32 °C, 150 r/min, 48 h). Cells were collected by centrifugation (13,000 r/min, 2 min), and bacterial genomic DNA was extracted using the TIANamp Bacteria DNA Kit (Tiangen Biotech, Beijing, China) following the manufacturer's instructions. The 16S rRNA gene was amplified using primers 27F (5′-AGAGTTTGATCCTGGCTCAG-3′) and 1492R (5′-TACGGCTACCTTGTTACGACTT-3′) under the following conditions: 95 °C for 5 min; 30 cycles of 95 °C for 30 s, 58 °C for 30 s, 72 °C for 1 min; and a final extension at 72 °C for 5 min.

#### 2.5.2 Yeasts

Purified yeasts were cultured in 5 ml of YPD broth at 30 °C, with shaking at 150 r/min for 48 hours. Cells were collected by centrifugation, and genomic DNA of yeast isolates was extracted using the Ezup Column Yeast Genomic DNA Purification Kit (Sangon Biotech, Shanghai, China) according to the manufacturer's instructions. The 26S rRNA gene was amplified with primers ITS1 (5′-TCCTCCGCTTATTGATATGC-3′) and ITS4 (5′-TCCGTAGGTGAACCTGCGG-3′). PCR conditions were as follows: initial denaturation at 94 °C for 5 min; 30 cycles of 94 °C for 1 min, 55 °C for 1 min, and 72 °C for 1 min; with a final extension at 72 °C for 5 min.

#### 2.5.3 Sequencing and phylogenetic analysis

PCR products were verified by agarose gel electrophoresis (1.5% agarose in 50 × TAE buffer, at 100 V for 30 min). Bands were visualized under UV light, and successful amplicons were sequenced commercially (Sangon Biotech, Shanghai, China) using the BigDye Terminator v3.1 Cycle Sequencing Kit (Applied Biosystems, Foster City, CA, USA) on an ABI 3730XL DNA Analyzer (Applied Biosystems, USA). The resulting sequences were compared with those in GenBank using BLAST, and phylogenetic trees were constructed using the Neighbor-Joining method implemented in MEGAX.

### 2.6 Microbial physiological and biochemical tests

#### 2.6.1 Growth curve

Strains preserved on solid slants were purified and inoculated into 5 ml of seed medium. For yeast isolates, the seed medium consisted of YPD broth (1% yeast extract, 2% peptone, 2% glucose, w/v). For lactic acid bacteria, MRS broth was used (1% peptone, 1% beef extract, 0.5% yeast extract, 2% glucose, 0.5% sodium acetate, 0.2% ammonium citrate, 0.02% magnesium sulfate, 0.005% manganese sulfate, w/v). For acetic acid bacteria, the seed medium contained glucose 5% (w/v), yeast extract 0.5% (w/v), and ethanol 1% (v/v). Cultures were incubated at 30 °C with shaking at 120 r/min for 12 h. The primary culture was then transferred into 50 ml of the same seed medium in 250 ml baffled flasks and incubated under identical conditions for 48 h. Samples were collected at regular intervals (in triplicate) to measure OD600, using a UV-6000 UV-Vis spectrophotometer (Shanghai Yuanxi Instruments Co., Ltd., Shanghai, China), and growth curves were plotted.

#### 2.6.2 Determination of optimal pH

For fermentation assays, the basal medium was prepared with black tea infusion (10 g of black tea leaves boiled in 1 L of distilled water for 10 min, then filtered) supplemented with 10% (w/v) sucrose as the primary carbon source. After cooling, the medium was sterilized by autoclaving at 121 °C for 15 min. Inoculated cultures were grown in 250 ml baffled flasks containing 100 ml of the fermentation medium, incubated at 30 °C with shaking at 120 r/min, the optimal pH was determined using a pH/ORP/temperature meter (HI2221, Shanghai JINGGONG Industrial Co., Ltd., Shanghai, China), uninoculated medium served as a control, OD600 was measured until a stable value was reached to determine the optimal pH for growth.

OD_600_ was measured directly using a UV-Vis spectrophotometer (UV-6000, Shanghai Yuanxi Instruments Co., Ltd., Shanghai, China) without dilution until a stable value was reached, to determine the optimal pH for growth, following standard methods for microbial growth monitoring [see Madigan et al. (9), Brock Biology of Microorganisms, 15th Edition, 2018].

### 2.7 Fermentation properties of black tea beverage produced with composite strains

#### 2.7.1 Preparation of sugared tea infusion

A tea infusion was prepared by adding 10 g of black tea leaves to 1.4 L of boiling water, followed by the addition of 10 g of sucrose. A total volume of 2.8 L of sugared tea infusion was obtained, homogenized, and sterilized by autoclaving at 121 °C for 15 min. For fermentation, 150 ml of sugared tea infusion was dispensed into each sterile bottle. Each treatment group was established with three independent fermentation bottles, which were considered as biological replicates. All subsequent analyses were conducted using these triplicate biological replicates.

#### 2.7.2 Activation of dominant strains

The dominant strains (JQ-1, JQ-2, CQF1, CQF2, RXA-1, and RXA-2) were reactivated at 28 °C and inoculated into a sugared tea infusion. Cultures were incubated in a rotary shaker at 28 °C with agitation at 180 rpm for 48 h. Cell concentrations were determined using a hemocytometer. Based on an initial suspension of approximately 1 × 10^6^ CFU/ml, serial dilutions were prepared to adjust all strains to comparable inoculation levels.

#### 2.7.3 Strain combination design

Each strain was cultured in its respective optimal medium until reaching an optical density of OD_600_ ≈ 1.0 (≈1 × 10^7^-10^8^ CFU/ml) before use: yeasts in YPD broth (1% yeast extract, 2% peptone, 2% glucose, w/v), acetic acid bacteria in glucose–yeast extract broth (5% glucose, 0.5% yeast extract, 1% ethanol, w/v/v), and lactic acid bacteria in MRS broth (composition as described in **Section 2.6.1**). More than 10 preliminary co-culture combinations were tested (data not shown), and the optimal assemblage of yeasts, acetic acid bacteria, and lactic acid bacteria was selected for further study. The final strain combinations are listed in Table 1. For each treatment, strains were co-inoculated at a total inoculation level of 4% (v/v), incubated at 30 °C.

**Table 1:** Reconfiguration of dominant bacteria in kombucha.

**ID**	**Strain combination method**	**Strain ratio**
A	JQ-1+JQ-2+CQF1+CQF2+RXA-1+RXA-2	1:1:1:1
B	JQ-1+JQ-2+CQF1+CQF2	1:1
C	JQ-1+JQ-2+RXA-1+RXA-2	1:1

#### 2.7.4 Determination of total phenol content

The total phenolic content (TPC) of kombucha samples was determined by the Folin–Ciocalteu colorimetric method (10–13). Briefly, 0.1 ml of sample was mixed with 10% Folin–Ciocalteu reagent and 7.5% sodium carbonate, diluted to 25 ml, and incubated at 45 °C for 15 min. Absorbance was measured at 765 nm. A standard curve was constructed using gallic acid (0–150 μg), and TPC was expressed as gallic acid equivalents. All analyses were performed in triplicate.

#### 2.7.5 DPPH radical scavenging ability

The free radical scavenging activity of kombucha was determined by the DPPH assay [(14), with modifications]. Briefly, 1 ml of sample supernatant was mixed with 4 ml of 50 μg/ml DPPH methanol solution and incubated in the dark at room temperature for 30 min. Absorbance was measured at 517 nm, with appropriate controls and blanks. DPPH scavenging activity (%) was calculated as:


$$\begin{array}{l} DPPH\;Scavenging\;Rate\;\left(\%\right)=\left[\frac{A_{o}-\left(A_{i}-A_{j}\right)}{A_{o}}\right]\times 100\% \end{array}$$

A_o_ = Absorbance of control (DPPH + distilled water)

A_i_ = Absorbance of sample (DPPH + sample)

A_j_ = Absorbance of sample background (sample + methanol)

#### 2.7.6 Determination of hydroxyl radical (·OH^−^) scavenging activity

The hydroxyl radical scavenging activity of kombucha was assessed following Jayabalan et al. (14) with modifications. In brief, 2 ml each of FeSO_4_ (9 mmol/L), ethanol–salicylic acid (9 mmol/L), deionized water, kombucha broth, and H_2_O_2_ (8.8 mmol/L) were combined and incubated at 37 °C for 30 min. A control mixture was prepared without H_2_O_2_. Absorbance was measured at 510 nm, and scavenging activity (%) was calculated relative to the control. All assays were conducted in triplicate.

Calculation of hydroxyl radical scavenging rate:


$$\begin{array}{l} OH^{-}ScavengingRate\left(\%\right)=\left[\frac{A_{o}-\left(A_{m}-A_{n}\right)}{A_{o}}\right]\times 100\% \end{array}$$

A_o_ = Absorbance of the control group (with H_2_O_2_ but without kombucha)

A_m_ = Absorbance of the sample group (with H_2_O_2_ and kombucha)

A_n_ = Absorbance of the sample background (without H_2_O_2_)

#### 2.7.8 Determination of total acid content

The total acid content of kombucha was measured through acid-base titration, following the method described by AOAC Official Method 942.15 (15). In brief, 1 ml of the sample was combined with phenolphthalein indicator and titrated with standardized 0.1 mol/L NaOH until a light pink endpoint appeared. A blank control solution consisting of deionized water with phenolphthalein was prepared and titrated under the same conditions to correct for any background alkalinity. The total acid content was then calculated and expressed as grams per liter of lactic acid using the specified formula.


$$\begin{array}{l} Totalacidcontent\left(g/L\right)=\frac{\left(V_{1}-V_{2}\right)\times C\times K\times 1,000}{V_{s}} \end{array}$$

V_1_: volume of NaOH consumed for sample titration (ml);

V_2_: volume of NaOH consumed in blank control (ml);

C: concentration of NaOH (mol/L);

K: molar mass of lactic acid (90.08 g/mol);

V_s_: volume of sample used (ml).

#### 2.7.9 Trace organic acids analysis

The organic acids in kombucha were analyzed using HPLC according to GB 5009.157-2016 and Ivanišová et al. (16). The separation was performed on a WONDASIL C18-WR column (5 μm, 4.6 × 150 mm) with a mobile phase of 0.1% phosphoric acid and methanol (97.5:2.5, v/v). The elution program included isocratic elution for 10 min, a gradient to 100% methanol for 5 min, and re-equilibration for 5 min. Conditions were: flow rate 0.6 ml/min, detection wavelength 210 nm, column temperature 37 °C, and injection volume 20 μl. Standard curves were prepared from tartaric, citric, succinic, and fumaric acids. Samples were diluted with ultrapure water, filtered through 0.45 μm membranes, and analyzed directly.

#### 2.7.10 Determination of total free amino acids content

The ninhydrin colorimetric method (17) was used to quantify free amino acids in kombucha. Standard curves were generated using glutamic acid, which reacted with phosphate buffer (pH 8.0) and 2% ninhydrin. The mixture was then heated in a boiling water bath for 15 min, and absorbance was measured at 570 nm. For sample analysis, 1 ml of kombucha was treated under the same conditions, and absorbance was measured at 570 nm against a reagent blank. All measurements were performed in triplicate.

#### 2.7.11 Determination of caffeine content

The caffeine content in kombucha was determined using high-performance liquid chromatography (HPLC), following the method described by Zhang et al. (18). A caffeine standard solution was used as a reference. Initially, 10 ml of kombucha was filtered through a 0.45 μm aqueous-phase filter membrane, and 5 ml of the filtrate was collected for analysis. HPLC analysis was performed using an ODS C18 column (150 mm × 4.6 mm, 4.5 μm) with a mobile phase consisting of methanol and water in a volume ratio of 20:80. The flow rate was kept at approximately 1.0 ml/min, the column temperature was set to 40 °C, and the detection wavelength was 273 nm. The injection volume was 20 μl. Before injection, both the standard caffeine solution and the sample filtrates were filtered through 0.45 μm membranes. The peak area of caffeine in the standard and the sample were measured and compared for quantification.

### 2.8 Sensory evaluation of kombucha solution

The sensory evaluation of kombucha was conducted using the 7-point hedonic scale. A panel consisting of 30 students and staff specializing in Food Science and Engineering from Wuyi University was recruited for the evaluation. In this study, only kombucha samples fermented for 8, 10, and 12 days were sampled for sensory analysis. Each panelist was required to evaluate the kombucha samples on the following sensory attributes: color, aroma, taste, texture, and overall acceptability.

### 2.9 Safety comparison between kombucha samples fermented via traditional back slopping method and using reconstituted fermentation cultures: spoilage microorganisms detection

Kombucha samples made through the traditional back-slopping method and those using reconstituted fermentation cultures were evaluated for safety. The focus was on detecting spoilage microorganisms.

#### 2.9.1 Traditional back-slopping kombucha

For the “back-slopping” condition, the inoculum for each new batch consisted of spent kombucha broth from the immediately preceding batch together with a fragment of the cellulosic pellicle (SCOBY). The inoculum was transferred directly into freshly prepared sweetened tea prepared under the same recipe and fermentation conditions as the reconstituted-culture experiments (A tea infusion was prepared by adding 10 grams of black tea leaves to 1.4 L of boiling water, followed by the addition of 10 grams of sucrose, incubated at 30 °C, under static aerobic conditions). Each transfer initiated a new batch without deliberate addition of exogenous microbes. Sampling for microbiology and chemistry was performed at matched time points to the reconstituted-culture condition, using aseptic technique.

#### 2.9.2 Spoilage microorganism detection by metagenomic sequencing

Total microbial DNA was extracted from kombucha samples using a bacterial DNA kit (Tiangen Biotech, Beijing, China) and a fungal DNA isolation kit (Norgen Biotek, Canada). The bacterial 16S rRNA V4–V5 region and fungal ITS regions were amplified with universal primers. Sequencing libraries were prepared with the Illumina TruSeq DNA kit and sequenced on the Illumina NovaSeq 6000 platform. Raw reads were filtered and clustered into OTUs at 97% similarity, and taxonomic assignment was performed using the SILVA database. Particular attention was paid to the detection of potential spoilage or pathogenic microorganisms, including *Candida, Aspergillus, Clostridium perfringens*, and *Burkholderia* species.

### 2.10 Data analysis

The data were analyzed using SPSS 19.0 software (IBM Corp., Chicago, USA) and Microsoft Excel 2016 (Microsoft Corp., Redmond, USA) for mapping purposes. Results were expressed as mean ± standard deviation. Statistical differences were evaluated by one-way analysis of variance (ANOVA) followed by Dunnett's test for multiple comparisons, and significance was set at *p* < 0.05 (95% confidence level).

Phylogenetic analysis was performed using MEGA 6.0 (19) with the neighbor–joining method, 1,000 bootstrap replicates, and the Kimura two-parameter model to estimate evolutionary distances.

## 3 Results and discussion

### 3.1 Isolation and identification of microorganisms

**Figure 1:**
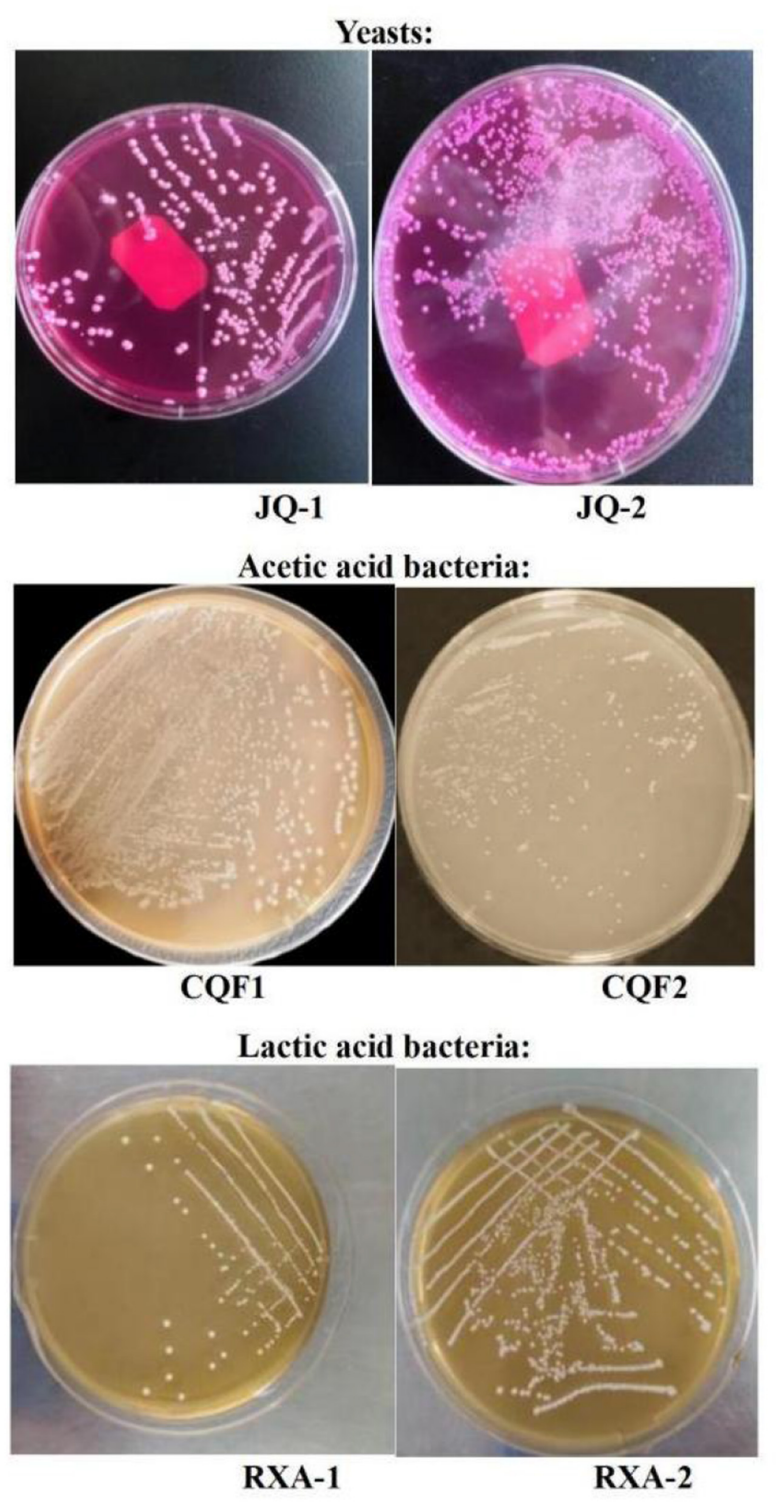
Colony morphology results of yeast, acetic acid bacteria, and lactic acid bacteria isolated from traditional kombucha fermentation broth.

Figure 1 presents the colony morphology and microscopic characteristics of the isolated microbial strains. The colony characteristics of JQ-1 were milky white, shiny, flat, with neat edges, and a large diameter. Microscopically, JQ-1 exhibited mostly round cell morphology with no spores. In contrast, JQ-2 colonies displayed a convex surface with fine granules, a wet and grayish-white appearance, and a slightly uneven edge. JQ-2 cells were predominantly oval and spore-free. The morphological characteristics of both JQ-1 and JQ-2 were consistent with typical yeast cells, as previously reported for kombucha-isolated yeasts such as *Saccharomyces, Zygosaccharomyces*, and *Lachancea fermentati*, which commonly exhibit round to oval, spore-free cell morphology and form shiny, milky-white to grayish colonies (6, 20, 21).

For the acetic acid bacteria, the colony characteristics of CQF1 were round, small in diameter, raised, white, with a wet and smooth surface. CQF2 colonies were similar, being round, small in diameter, raised, white, with neat and smooth edges. Gram staining showed that both CQF1 and CQF2 cells were Gram-negative, appearing red under the microscope. They exhibited a rod-shaped morphology and were arranged in pairs, chains, or as single cells. These characteristics aligned with the known morphology of acetic acid bacteria previously reported in kombucha studies (8, 22, 23).

The colonies of RXA-1 and RXA-2 were round, milky white, small in diameter, raised, and had a wet surface. Gram staining indicated that both RXA-1 and RXA-2 were Gram-positive, staining blue under the microscope. They exhibited long rod-like shapes and were spore-free. These features were consistent with the known morphology of lactic acid bacteria previously described in kombucha microbiota studies (24, 25, 47).

The observed differences in colony and cell morphology among strains within the same microbial group suggest potential functional diversity. In this project, two distinct strains for each type of microorganism (yeast, AAB, and LAB) were reconstituted as starter SCOBY, with each functional group adjusted to an initial level of approximately 10^6^ CFU/ml, to ensure a balanced and robust fermentation process. This microbial diversity within the SCOBY is thought to drive variations in metabolic activity, thereby shaping the flavor profile, acidity, and probiotic potential of the final kombucha product.

### 3.2 PCR amplification and gel electrophoresis analysis

The PCR amplification of the isolated microbial strains from kombucha was performed to confirm their genetic identity and purity. The DL2000 DNA marker (M) was used as a reference, and a and a non-template control (NTC) was included to rule out contamination during the PCR process.

The PCR products of the yeast strains exhibited distinct and well-defined bands, indicating successful amplification of the target gene regions. JQ-1 displayed a band size consistent with its expected fragment length (i.e., 750 bp), whereas JQ-2 produced a similar, though slightly varied band, suggesting potential strain-level genetic diversity (8, 20). This diversity is crucial for fermentation dynamics, as different yeast strains can contribute varied metabolic profiles influencing flavor, aroma, and fermentation efficiency, as demonstrated in previous kombucha studies (26).

For the acetic acid bacteria isolates CQF1 and CQF2, both exhibited clear but slightly smeared bands at approximately 1,500 bp. This band size corresponds to the expected length of the 16S rRNA gene for bacterial isolates in general, and confirms successful amplification of the acetic acid bacteria strains (6, 27). Despite their similar band positions, these products could uncover genetic variations responsible for differences in acetic acid production rates, which directly affect the acidity and preservation quality of kombucha.

The lactic acid bacteria also showed well-defined bands for RXA-1 and RXA-2. The identical band positions observed for isolates RXA-1 and RXA-2 at ~1.5 kb (≈1,450–1,500 bp) indicate successful amplification of the bacterial 16S rRNA gene and are consistent with lactic acid bacteria (LAB). While gel band size alone is not diagnostic at the group level, the electrophoretic pattern, together with LAB-typical phenotypes—Gram-positive staining, catalase negativity, robust growth on MRS at 37 °C, and medium acidification—supports their assignment to the LAB guild. However, sequencing is necessary to determine strain-specific traits such as varying probiotic potential, antimicrobial activity, and contributions to kombucha's functional properties.

The absence of any bands in the non-template control (NTC) indicated that no contamination was introduced during PCR amplification or electrophoresis, thereby confirming the integrity of the experimental setup.

Based on the gel electrophoresis results, all isolates yielded amplicons of the expected size for 16S rRNA (bacteria) or ITS (yeasts), confirming successful PCR amplification. While these results do not by themselves establish the purity of the isolates, the combination of repeated subculturing, colony morphology assessment, microscopic observation, and subsequent sequencing provided evidence for the successful isolation of representative yeast, acetic acid bacteria, and lactic acid bacteria strains. The distinct banding at the expected sizes supports their classification, which was further validated through phylogenetic analysis.

### 3.3 Genetic characterization and phylogenetic analysis

**Figure 2:**
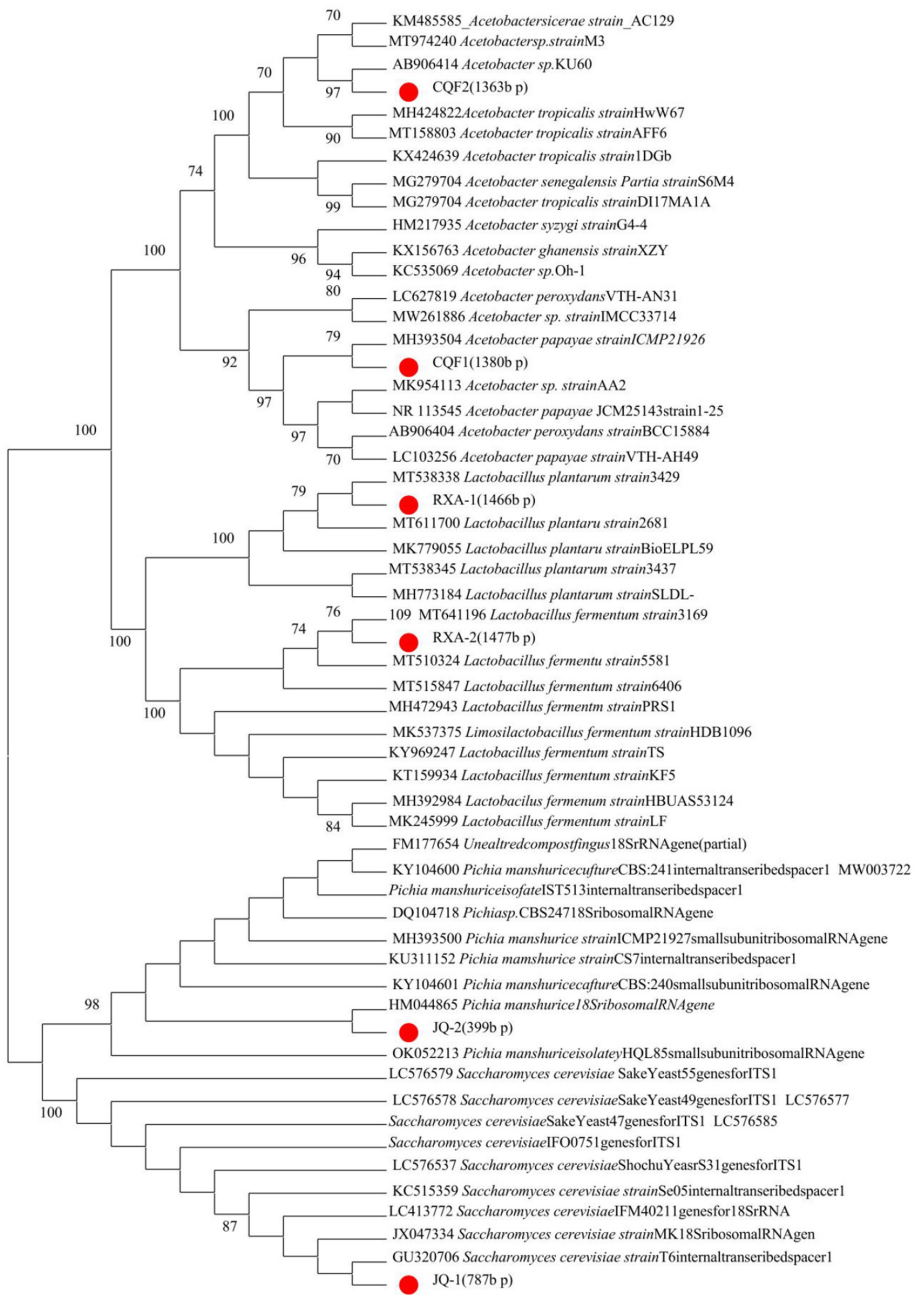
Phylogenetic trees of yeasts, acetic acid bacteria, and lactic acid bacteria isolated from kombucha, based on ITS (yeasts) and 16S rRNA (bacteria) gene sequences. Bootstrap values (1,000 replicates) are shown at the nodes.

Figure 2 presents the phylogenetic tree constructed from gene sequencing data of dominant microbial strains isolated from traditional kombucha fermentation broth. This tree highlights the genetic relationships among yeast (A: JQ-1, JQ-2), acetic acid bacteria (B: CQF1, CQF2), and lactic acid bacteria (C: RXA-1, RXA-2) in comparison to established reference strains.

The gene sequencing results provide crucial insights into the genetic characterization of the isolated strains, confirming their identity and evolutionary relationships with well-established species. The yeast isolates JQ-1 and JQ-2 clustered closely with *Saccharomyces cerevisiae* and *Pichia manshurica*, respectively, indicating their affiliation with this widely recognized fermentative yeast known for its robust metabolic activity in kombucha fermentation (8, 26).

The acetic acid bacteria strains CQF1 and CQF2 exhibited close genetic alignment with *Acetobacter* species, specifically aligning with *Acetobacter papayae* and *Acetobacter tropicalis*. These species are well-known for their role in acid production, defining characteristics of kombucha's acidic and tangy profile (22, 27). The genetic proximity of these strains suggests their ability to produce acetic acid efficiently, supporting the preservation and flavor development of kombucha.

The lactic acid bacteria strains RXA-1 and RXA-2 were identified as closely related to *Lactobacillus plantarum* and *Lactobacillus fermentum*, both of which are recognized for their probiotic potential and contribution to the functional properties of fermented foods (6, 28). Their presence may enhance kombucha's health benefits by providing beneficial bacteria that support gut health and modulate the microbial ecosystem.

The successful isolation, purification, and genetic characterization of these strains lay a strong foundation for further functional analysis and application in controlled kombucha production systems.

### 3.4 Biochemical determination of isolated microorganisms

#### 3.4.1 Growth curve analysis

**Figure 3:**
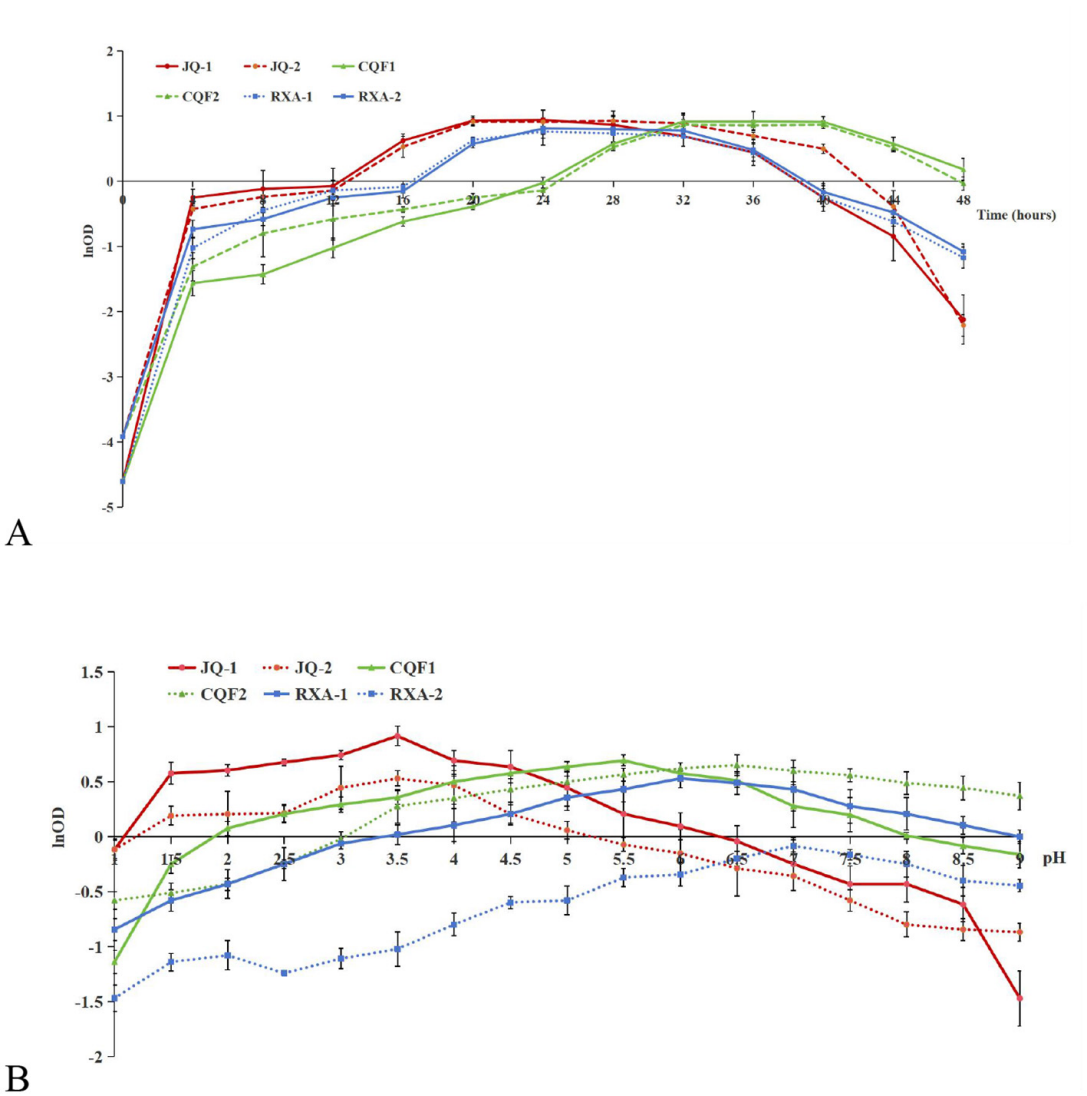
Growth response of dominant yeast and bacterial strains isolated from traditional kombucha fermentation broth as a function of fermentation time **(A)** and pH **(B)**. *Saccharomyces cerevisiae* (JQ-1), *Pichia manshurice* (JQ-2), *Acetobacter papaya* (CQF1) and *Acetobacter tropicalis* (CQF2), *Lactobacillus plantarum* (RXA-1) and *Lactobacillus fermentum* (RXA-2). Data represent mean ± SD of three biological replicates.

The growth curve analysis of dominant bacterial and yeast strains isolated from traditional kombucha fermentation broth reveals distinct proliferation dynamics and metabolic activities over a 48 h period (Figure 3A). The yeast strains JQ-1 and JQ-2 exhibited a rapid increase in cell density during the first 12 h, reaching their maximum optical density (OD_600_) at approximately 24 h. They maintained a stable phase until 32 h and then declined sharply after 36 h. Quantitative analysis of the exponential phase indicated maximum specific growth rates (μmax) of 0.096 h^−1^ for JQ-1 and 0.103 h^−1^ for JQ-2, suggesting that JQ-2 proliferated slightly faster, while JQ-1 displayed a more stable growth trajectory. These differences likely reflect variations in metabolic capacity or tolerance to fermentation by-products.

The acetic acid bacterial strains CQF1 and CQF2 showed slower initial growth, followed by a significant increase after 20 h. Both strains reached their growth peaks between 32 and 36 h. Their μmax values were 0.113 h^−1^ (CQF1) and 0.100 h^−1^ (CQF2), which was consistent with their delayed but efficient proliferation once ethanol became available.

This pattern corresponds to their metabolic role of oxidizing ethanol—produced by yeast—into acetic acid, which contributes to the characteristic acidity and flavor of kombucha. Although ethanol and acetic acid concentrations were not directly measured in this study, this growth trend is consistent with previous findings that acetic acid bacteria dominate the mid- to-late fermentation stages (29–31).

The lactic acid bacterial strains RXA-1 and RXA-2 exhibited moderate and sustained growth, attaining their maximum cell density between 28 and 32 h, followed by a gradual decline after 40 h. Their μmax values were 0.086 h^−1^ and 0.098 h^−1^, respectively. This implies that lactic acid bacteria are predominantly active during the late exponential to stationary phases.

The growth and pH tolerance profiles of the isolates highlight their distinct ecological roles during kombucha fermentation (Figure 3B). The yeast strains JQ-1 and JQ-2 exhibited rapid proliferation over a wide pH range, maintaining significant growth from pH 2.5 to 6.0, with optimal cell densities near pH 3.0–3.5. Their maximum specific growth rates (μ_max_) were 0.096 h^−1^ and 0.103 h^−1^, respectively. The strong acid tolerance of these yeasts supports their essential role in the early fermentation phase, where they metabolize sugars to produce ethanol and CO_2_ under acidic conditions.

The acetic acid bacterial strains CQF1 and CQF2 displayed moderate pH adaptability, sustaining growth between pH 3.5 and 7.0, and achieving μ_max_ values of 0.113 h^−1^ (CQF1) and 0.100 h^−1^ (CQF2). Their optimal growth occurred near pH 5.0–6.0, aligning with their metabolic capacity to oxidize yeast-derived ethanol into acetic acid. This activity is crucial for increasing kombucha's acidity and developing its characteristic flavor.

In contrast, the lactic acid bacteria RXA-1 and RXA-2 showed reduced tolerance to low pH, exhibiting optimal growth between pH 6.0 and 7.0, and declining sharply under pH < 4.0. Their μmax values were 0.086 h^−1^ and 0.098 h^−1^, respectively. This restricted acid tolerance suggests that lactic acid bacteria become metabolically active during later fermentation stages, where they help buffer excessive acidity and contribute to flavor refinement and functional metabolite production.

Taken together, these results demonstrate that microbial diversity, with yeasts initiating fermentation, acetic acid bacteria intensifying acidification, and lactic acid bacteria stabilizing the final beverage, is crucial for maintaining process stability, product consistency, and desirable sensory outcomes (8, 24).

### 3.5 Functional properties of kombucha fermented with a reconstituted fermentation culture

#### 3.5.1 Total phenol content

**Figure 4:**
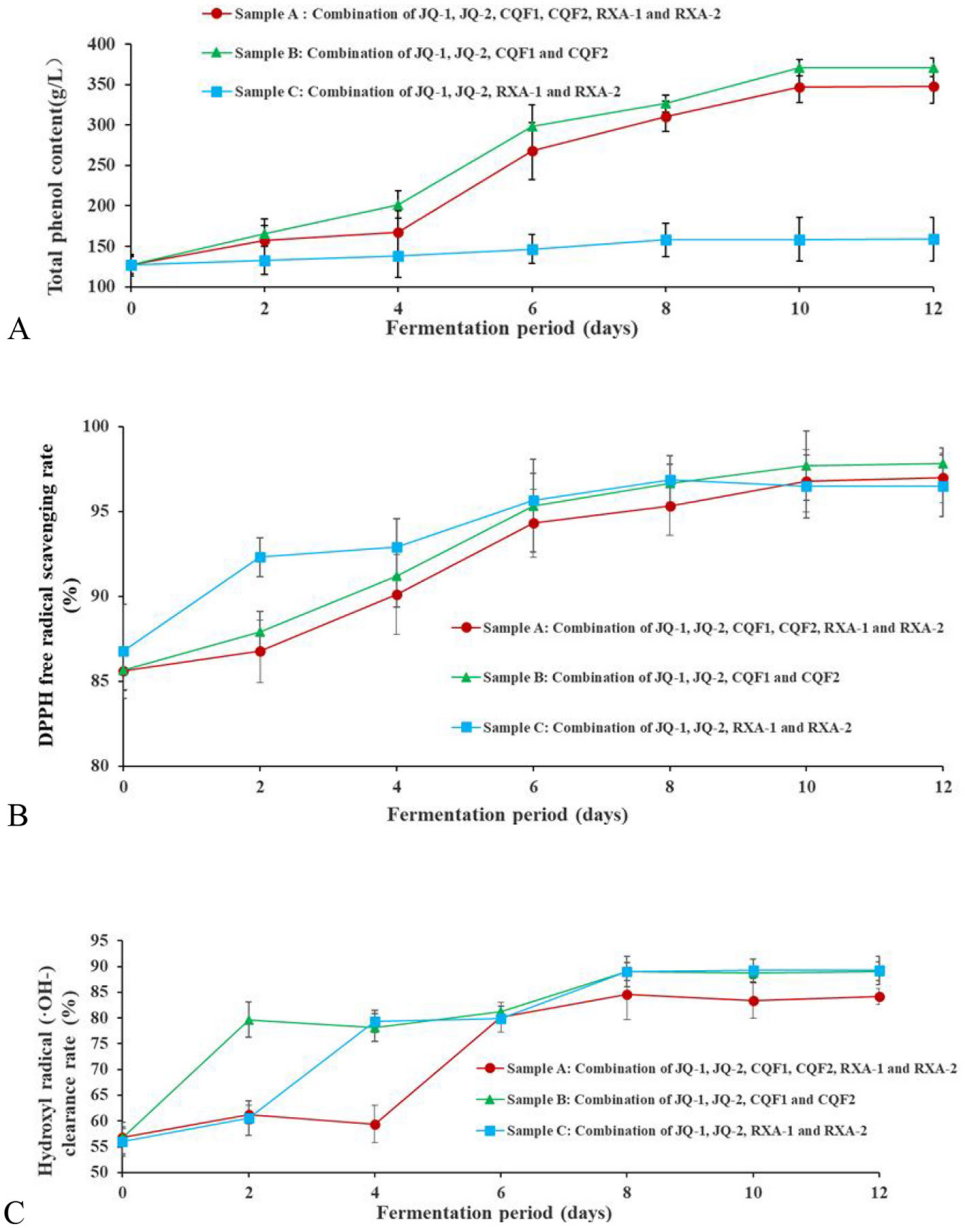
Changes in total phenol content **(A)**, DPPH radical scavenging rate **(B)** and hydroxyl radical clearance rate **(C)** of kombucha fermented using black tea by recombined SCOBY over a 12-day fermentation period. *Saccharomyces cerevisiae* (JQ-1), *Pichia manshurice* (JQ-2), *Acetobacter papaya* (CQF1) and *Acetobacter tropicalis* (CQF2), *Lactobacillus plantarum* (RXA-1) and *Lactobacillus fermentum* (RXA-2). Data represent mean ± SD of three biological replicates.

Phenols are a group of substances with strong antioxidant properties. They are widely used as antioxidants in the food, healthcare, pharmaceutical, and cosmetic industries. In kombucha, phenols are the main compounds responsible for its characteristic astringency and bitter taste. As depicted in Figure 4A, the total phenol content of kombucha increased after fermentation with different microbial strain combinations compared to pre-fermentation levels. Sample A (combining JQ-1, JQ-2, CQF1, CQF2, RXA-1, and RXA-2) reached a peak total phenol content of 370.49 ± 1.32 g/L on the 10th day, while Sample B (combining only JQ-1, JQ-2, CQF1, and CQF2) showed a slightly lower value of 347.00 ± 1.73 g/L on the 10th day. In contrast, Sample C (combining only JQ-1, JQ-2, RXA-1, and RXA-2) exhibited minimal overall change, with a maximum value of 158.00 ± 2.66 g/L on the 8th day. Polyphenols in black tea are widely recognized for their potent antioxidant properties (17, 32–34). In the present study, the total polyphenol concentrations exhibited a progressive increase throughout the fermentation process, which is consistent with findings from more recent research (35, 36). These studies highlight the dynamic changes in polyphenol content during fermentation, suggesting that microbial interactions play a significant role in the transformation and release of bioactive polyphenols, contributing to the antioxidant activity of kombucha.

The observed increase in polyphenol content may be attributed to the enzymatic activity of β-glucosidase, cellulase, and pectinase, as well as the organic acids released by bacteria and yeast during kombucha fermentation. These enzymes likely break down tea tissues, leading to the release of bound phenols. Moreover, the transformation of large phenolic molecules into smaller, more bioavailable phenols under enzymatic action further contributes to the observed increase in polyphenol content (11).

Previous studies have shown similar mechanisms. For example, cellulase and esterase produced by microorganisms were found to release bound phenols from rice bran after fermentation (37). Ivanisova et al. (12) examined black tea-based kombucha and discovered that the total polyphenol content increased significantly after fermentation, which was attributed to the enzymatic hydrolysis of bound phenolic compounds.

During kombucha fermentation using the three different strain combination methods, the raw materials and fermentation conditions remained consistent, with only the strain compositions varying. This suggests that the synergistic interactions between different microbial strains played a crucial role in the generation and release of total phenols.

#### 3.5.2 DPPH scavenging activity

Figure 4B shows the DPPH free radical scavenging activity of kombucha made with recombined strains over 12 days. The DPPH (2,2-diphenyl-1-picrylhydrazyl) radical scavenging test is widely used to measure antioxidant activity, reflecting a compound's ability to donate hydrogen and neutralize free radicals.

As depicted, the DPPH scavenging rate increased progressively with fermentation time across all strain combinations (Samples A, B, and C). In Sample A, the rate consistently remained above 85.00% throughout the process. Meanwhile, Sample B exhibited a steady increase, reaching a peak of 97.69 ± 0.06% on day 10. Sample C followed a similar upward trend, attaining 96.47 ± 0.08% on day 10. After the 10th day, the scavenging rates in all groups plateaued.

A comparative analysis revealed that Sample B demonstrated the highest activity, indicating superior antioxidant potential. These findings confirm that fermentation significantly enhances the antioxidant capacity compared to the unfermented tea. However, the variations among strain combinations reflect differences in microbial metabolism.

The observed increase in antioxidant activity corresponds to the rise in total phenolic content (Figure 4A), suggesting that phenolic compounds play a central role in radical scavenging. The enzymatic activity of yeast and bacteria likely contributes by releasing bound phenols and converting complex polyphenols into more bioavailable forms. This mechanism is consistent with previous reports (12, 14).

More recent studies have also confirmed that microbial fermentation enhances the antioxidant properties of kombucha (35, 36, 38). The subtle differences observed between strain combinations imply that microbial synergy, particularly the balance among yeast, acetic acid bacteria, and lactic acid bacteria, influences the extent of phenolic release and transformation. The superior performance of Sample B may therefore reflect a more efficient interaction of enzymatic activity and metabolic by-products, resulting in higher scavenging capacity.

#### 3.5.3 Hydroxyl radical scavenging capacity

The reducing power of a beverage indicates its capacity to donate electrons to other compounds, thereby reflecting its potential antioxidant activity (39). Figure 4C shows the hydroxyl radical (·OH–) scavenging ability of black tea fermented by various reconstituted cultures over 12 days. Hydroxyl radicals are highly reactive species that cause oxidative damage, and their neutralization is an important marker of antioxidant potential.

The hydroxyl radical scavenging ability of Samples A and B exhibited a similar dynamic pattern, with an initial increase from day 0 to 2, a slight decrease between days 2 and 4, followed by a steady rise until day 8, and a subsequent decline from day 8 to 12. The highest scavenging activities were 84.62 ± 1.69% for Sample A and 89.00 ± 1.00% for Sample B on day 8. In contrast, Sample C showed a continuous increase in hydroxyl radical scavenging capacity from day 0 to 8, reaching a peak of 89.00 ± 1.32%, but then declined after day 8. The initial scavenging activity was largely derived from tea components, with the early increase attributed to the release of tea-derived antioxidants. The later decline may result from nutrient depletion in the fermentation medium, which restricts microbial growth and reduces metabolic activity, thereby limiting the production of antioxidant metabolites. These fluctuations suggest that both microbial metabolism and substrate availability (e.g., polyphenols, sugars, and amino acids serving as precursors for antioxidant compounds) are crucial in shaping the antioxidant profile of kombucha. Moreover, the differences observed among strain combinations underscore the role of microbial composition in determining the efficiency of antioxidant production and the release of bioactive compounds.

#### 3.5.4 Total acid content

**Figure 5:**
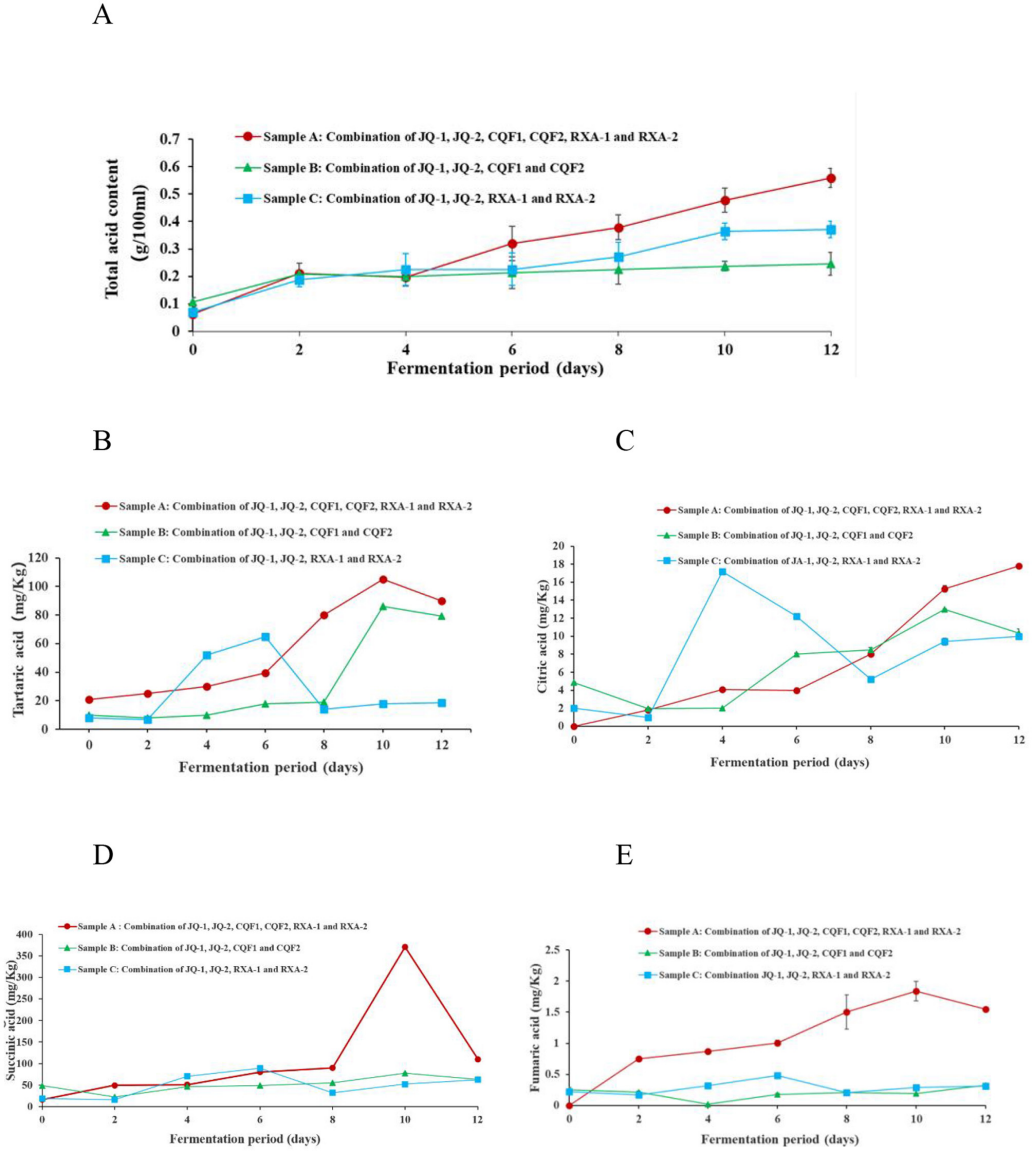
Changes in **(A)** total acid content, **(B)** tartaric acid, **(C)** citric acid, **(D)** Succinic acid, and **(E)** fumaric acid of kombucha fermented using black tea by recombined SCOBY over a 12-day of fermentation period. *Saccharomyces cerevisiae* (JQ-1), *Pichia manshurice* (JQ-2), *Acetobacter papaya* (CQF1) and *Acetobacter tropicalis* (CQF2), *Lactobacillus plantarum* (RXA-1) and *Lactobacillus fermentum* (RXA-2). Data represent mean ± SD of three biological replicates.

As shown in Figure 5A, the total acid content in the fermentation broth of Sample A consistently increased from day 0 to 12, showing the highest overall rate of increase. Sample C showed a similar trend but increased at a lower rate. It is worth noting that Sample C increased total acid content from day 0 to 2 and then leveled off, exhibiting the slowest rate of increase overall.

The continuous increase in acidity during fermentation reflects the metabolic activity of the microbial strains involved, particularly through the production of primary organic acids such as acetic acid, gluconic acid, and lactic acid (3, 14, 36). Variations in acid production rates among different strain combinations are likely due to differences in metabolic efficiency and enzymatic capabilities. From a safety standpoint, it is crucial that sufficient acid is produced to lower the pH of kombucha below 4.6 within the first 0–4 days of fermentation, effectively inhibiting the growth of spoilage and pathogenic microorganisms. However, the final total acid content of the kombucha ultimately affects its overall consumer acceptability (36, 40).

#### 3.5.5 Trace organic acids analysis

This study examined specific trace organic acids—tartaric, citric, succinic, and fumaric—due to their potential influence on flavor development and their dynamic changes during fermentation, which have not been well-documented. While acetic acid and gluconic acid are well-established as the primary organic acids in kombucha, the variation and behavior of these lesser-studied acids offer new insights into microbial activity and metabolic interactions. Previous studies have comprehensively reported acetic, gluconic, and lactic acid as dominant acids in kombucha (29, 41).

Figure 5 shows the changes in four organic acids—tartaric (Figure 5B), citric (Figure 5C), succinic (Figure 5D), and fumaric (Figure 5E)—during black tea fermentation using three different reconstituted cultures (Samples A, B, and C). These organic acids are important not only for their contribution to the sourness and overall flavor profile of kombucha but also for their role in promoting health benefits such as improved digestion and microbial balance (3, 36, 42).

In Sample A, tartaric acid, succinic acid, and fumaric acid gradually increased from day 0 to 10, reaching their highest levels on the 10th day before slightly declining, while citric acid consistently rose throughout the 12-day fermentation, with pronounced increases on days 6 and 12, peaking at 17.88 ± 0.34 mg/kg. Sample B displayed greater variability: tartaric acid increased steadily to a maximum of 84.16 ± 0.72 mg/kg on day 10, fumaric acid decreased during the first 4 days but rose sharply thereafter to 0.33 ± 0.02 mg/kg on day 12, and succinic acid and citric acid both peaked on day 10 at 75.00 ± 0.26 mg/kg and 15.00 ± 0.52 mg/kg, respectively. In Sample C, organic acid profiles were more fluctuating, with marked decreases on days 2 and 6 followed by an upward trend from day 8 to 12; tartaric acid (61.91 ± 0.08 mg/kg), succinic acid (88.94 ± 0.21 mg/kg), and fumaric acid (0.48 ± 0.01 mg/kg) reached their maxima on day 6 before declining, while citric acid fluctuated, peaking on day 4 at 16.30 ± 0.40 mg/kg.

Tartaric acid, generally stable in tea, may accumulate through microbial transformation of polyphenolic precursors and decline when further metabolized. Succinic acid is a well-known by-product of the tricarboxylic acid (TCA) cycle in yeast and acetic acid bacteria, explaining its accumulation during active growth and reduction as fermentation slows. Fumaric acid, also an intermediate of the TCA cycle, tends to accumulate later in fermentation, consistent with microbial adaptation to nutrient stress. Citric acid fluctuations, particularly in Sample C, can be attributed to early release or synthesis followed by microbial utilization, especially by lactic acid bacteria via citrate metabolism. Collectively, these patterns highlight distinct fermentation dynamics among the strain combinations, with different metabolic pathways contributing to acid accumulation or depletion. Such differences not only shape the sourness and flavor complexity of kombucha but may also affect its functional properties, emphasizing the importance of microbial composition in determining fermentation outcomes (3, 30, 31).

#### 3.5.6 Free amino acid content changes

**Figure 6:**
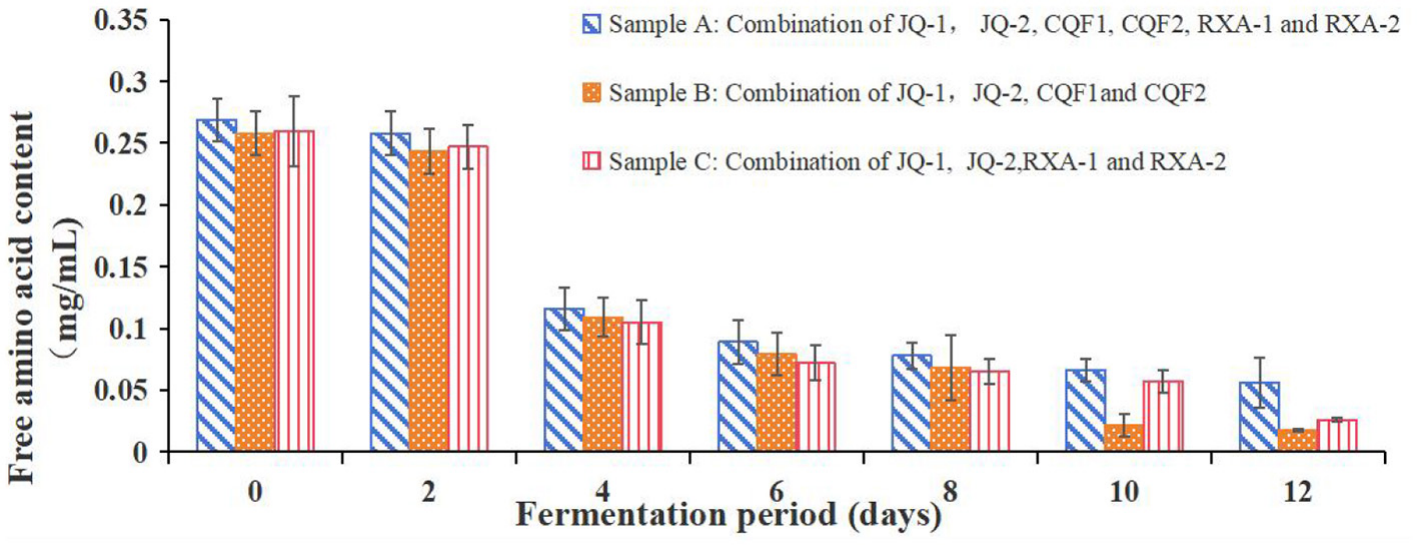
Free amino acid content of kombucha fermented using black tea and recombined SCOBY over a 12-day fermentation period. *Saccharomyces cerevisiae* (JQ-1), *Pichia manshurice* (JQ-2), *Acetobacter papaya* (CQF1) and *Acetobacter tropicalis* (CQF2), *Lactobacillus plantarum* (RXA-1) and *Lactobacillus fermentum* (RXA-2). Data represent mean ± SD of three biological replicates.

Figure 6 shows the free amino acid content of black tea fermented by the recombined strains over 12 days. Free amino acids are essential contributors to the taste, nutritional value, and metabolic activity of kombucha. They act as precursors for various flavor compounds and play a crucial role in microbial growth and fermentation efficiency.

As illustrated in Figure 6, the free amino acid content of all strain combinations (A, B, and C) showed a general downward trend throughout the fermentation process. At day 0, the initial free amino acid content was relatively high for all combinations, with values around 0.265 ± 0.005 mg/ml. The concentrations of free amino acids rapidly decreased during the first 4 days and remained at a low level (< 0.1 mg/ml) after that.

The decline in free amino acid content can be attributed to their role as the primary nitrogen source for microbial growth, as no supplemental nitrogen source was added during kombucha fermentation. This is consistent with findings from Škraban and Trček (43) and Tran et al. (44), who reported similar trends in other fermentation studies. The consistent reduction suggests that amino acids were actively utilized by microorganisms for the synthesis of proteins, enzymes, and other metabolic compounds.

Among the three samples, Sample A maintained slightly higher levels of free amino acids compared to Samples B and C throughout the fermentation period. This may indicate differences in microbial metabolism and efficiency in utilizing nitrogen sources.

This suggests that amino acids are likely being converted into metabolic byproducts that contribute to the complex flavor profile of kombucha. These findings underscore the dynamic interplay between microbial activity and nutrient utilization in shaping the biochemical characteristics of fermented kombucha.

#### 3.5.7 Caffeine content changes

**Figure 7:**
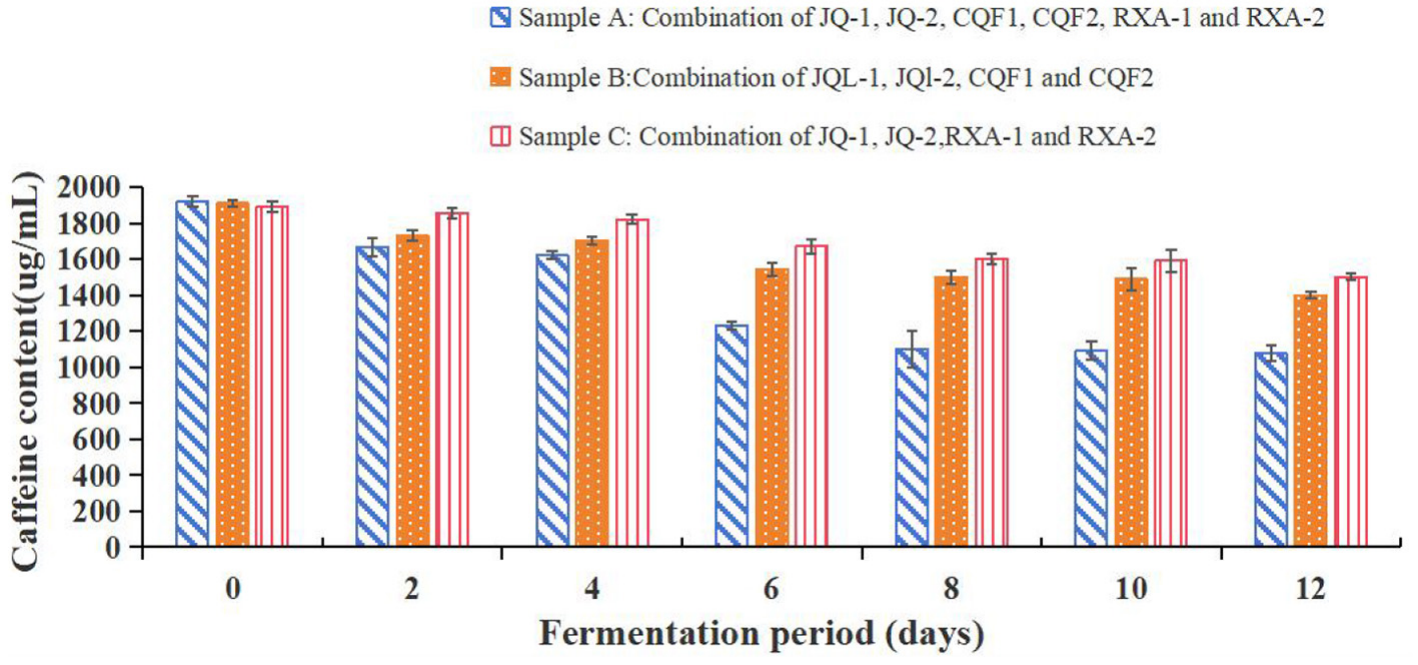
Caffeine content of kombucha fermented using black tea and recombined SCOBY over a 12-day fermentation period. *Saccharomyces cerevisiae* (JQ-1), *Pichia manshurice* (JQ-2), *Acetobacter papaya* (CQF1) and *Acetobacter tropicalis* (CQF2), *Lactobacillus plantarum* (RXA-1) and *Lactobacillus fermentum* (RXA-2). Data represent mean ± SD of three biological replicates.

Figure 7 illustrates the caffeine content of black tea fermented by the recombined strains over 12 days. Caffeine is one of the key bioactive compounds in tea-based beverages, contributing to their stimulating effects and characteristic bitterness.

As depicted in Figure 7, the caffeine content in all three strain combinations (A, B, and C) decreased progressively over the fermentation period. On day 0, the initial caffeine content was around 2,000 ng/ml for all samples. By day 12, sample A's caffeine content had decreased to about 1,200 ng/ml, while samples B and C had slightly higher levels.

The gradual decline in caffeine concentration during fermentation is consistent with previous studies, which suggest that microbial metabolism contributes to the breakdown or transformation of caffeine. This could be attributed to specific microbial enzymes that can demethylate or degrade caffeine into other metabolic products (29).

Among the three samples, sample A showed a more significant reduction in caffeine content compared to samples B and C. This could indicate differences in the metabolic activity of the microbial consortia involved, with sample A's combination of strains potentially having higher caffeine-degrading capabilities.

The reduction in caffeine content aligns with changes observed in other metabolic parameters like organic acids and free amino acids, reflecting the dynamic biochemical transformations occurring throughout the fermentation process. This decrease in caffeine may also impact the sensory profile of the final kombucha, potentially reducing its bitterness and altering its stimulant properties.

#### 3.5.8 Sensory analysis

**Figure 8:**
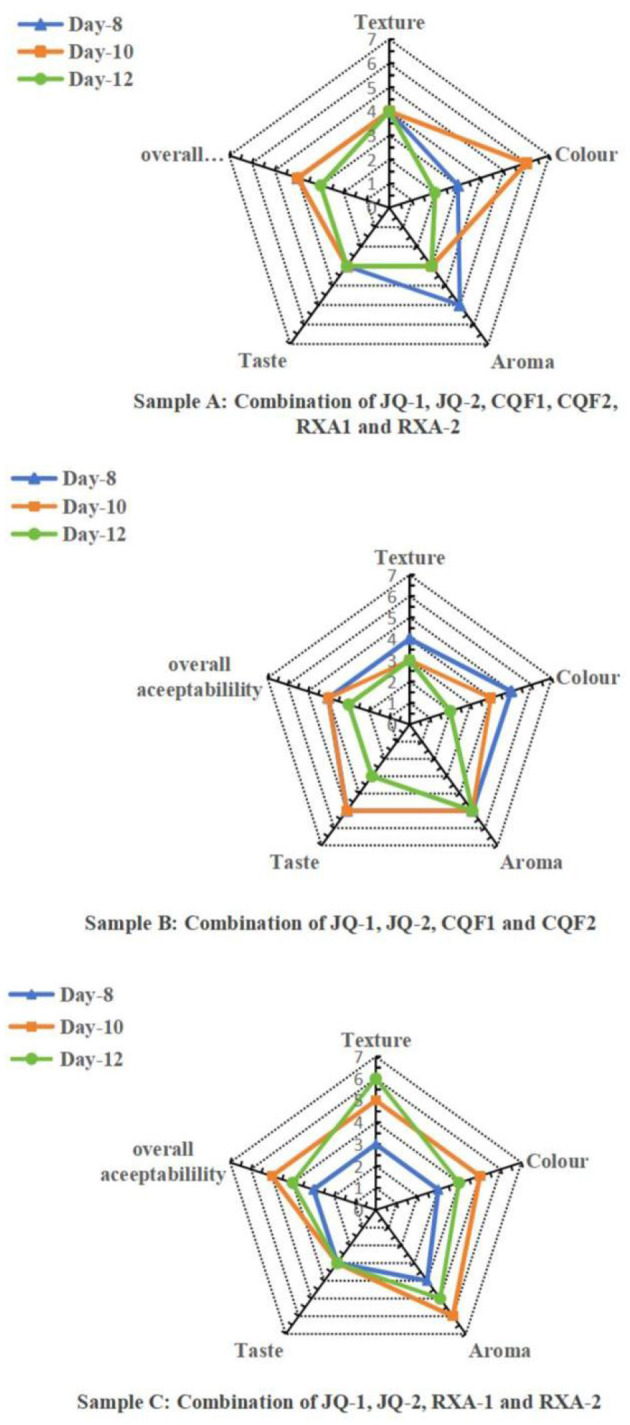
Radar plots representing sensory evaluation of kombucha samples fermented for, 8, 10 and 12 days.*Saccharomyces cerevisiae* (JQ-1), *Pichia manshurice* (JQ-2), *Acetobacter papaya* (CQF1) and *Acetobacter tropicalis* (CQF2), *Lactobacillus plantarum* (RXA-1) and *Lactobacillus fermentum* (RXA-2). Data represent mean ± SD of three biological replicates.

Sensory evaluation was conducted on Samples A, B, and C fermented for 8, 10, and 12 days. The sensory attributes evaluated included color, texture, aroma, taste, and overall acceptability rated on a 7-point hedonic scale. The radar plots in Figure 8 illustrate distinct trends across fermentation periods and microbial combinations.

Sample A (a combination of *Saccharomyces cerevisiae, Pichia manshurice, Acetobacter papayae, Acetobacter tropicalis, Lactobacillus plantarum* and *Lactobacillus fermentum*) exhibited the most balanced sensory profile on day 10, achieving the highest scores for aroma, taste, and overall acceptability. Although the texture score slightly decreased from day 8 to 12, it remained ≥4.0/6.0 and was not significantly different from day 10, indicating that texture remained within the pre-specified acceptability range. Notably, the day 12 sample experienced a reduction in aroma and taste scores, indicating that prolonged fermentation might have increased acidity, negatively affecting palatability. This sample benefits from the synergistic activity of all three microbial groups, particularly around day 10, which seemed to enhance flavor complexity and consumer preference.

Sample B (a combination of *Saccharomyces cerevisiae, Pichia manshurice, Acetobacter papayae* and *Acetobacter tropicalis*) lacked lactic acid bacteria and displayed a different sensory trajectory. Day 8 received the highest scores for most attributes, especially in texture and taste, suggesting a more favorable product early in fermentation. However, sensory scores progressively declined by day 12, the text now states that the decline was primarily in taste and texture, while aroma remained stable. This pattern underscores the importance of lactic acid bacteria in modulating sensory outcomes, possibly by contributing mild acidity and smoothing flavor.

Sample C (a combination of *Saccharomyces cerevisiae, Pichia manshurice, Lactobacillus plantarum* and *Lactobacillus fermentum*), which excluded acetic acid bacteria, performed consistently throughout the fermentation days, with only minor variations among attributes. The day 10 sample achieved the highest overall acceptability, On day 10, aroma and taste were well-rated (≥4.5/6.0). Texture and color also remained relatively stable across all days, indicating a less dynamic fermentation profile, this is likely due to the absence of acetic acid bacteria, which are known for contributing to volatile acidity and tanginess.

Overall, the 10-day fermentation period yielded the most favorable sensory outcomes for Samples A and C, suggesting that this duration allows for optimal microbial interaction and flavor development without excessive acid buildup. In contrast, Sample B was more appealing on day 8, reflecting the limitations of fermentations lacking lactic acid bacteria. These findings emphasize the importance of carefully selecting microbial combinations and fermentation duration to produce a kombucha product that balances safety, quality, and consumer preference.

### 3.6 Safety comparison between kombucha samples fermented via traditional back slopping method and using reconstituted fermentation cultures: spoilage microorganisms detection

From a microbiological safety perspective, metagenomic analysis revealed a variety of fungal and bacterial pathogens in back-slopping kombucha samples. These included fungal such as *Candida saopaulonensis, Fusarium* spp., *Malassezia arunalokei*, and several clinically relevant bacterial taxa such as *Acinetobacter* spp., *Burkholderia* spp., *Clostridium perfringens*, and *Mycobacterium mucogenicum*. These microorganisms are linked to spoilage, toxin production, and potential health risks. In contrast, none of these pathogens were detected in the customized SCOBY-fermented kombucha samples, suggesting that the reconstituted fermentation cultures can effectively suppress or exclude undesirable contaminants during fermentation.

Collectively, these findings underscore that customized microbial fermentation not only enhances the stability and reproducibility of kombucha production but also provides significant food safety advantages by reducing the risk of contamination with pathogenic microorganisms.

## 4 Conclusions

Building on our previous work that established these six strains (*Saccharomyces cerevisiae, Pichia manshurice, Acetobacter papayae, Acetobacter tropicalis, Lactobacillus plantarum*, and *Lactobacillus fermentum*) as dominant members of traditional kombucha communities. This study successfully isolated six dominant microbial strains from traditional kombucha and reconstructed them into defined consortia for controlled fermentation. The reconstituted cultures exhibited a favorable/characteristic biochemical and functional profile during fermentation functional and biochemical properties, including increased phenolic content, improved antioxidant capacity, reduced caffeine, and balanced organic acid profiles. Sensory evaluation highlighted that consortia containing both yeasts and acetic acid bacteria achieved the highest acceptability. Importantly, DNA-based metagenomic analysis showed that no reads above the reporting threshold were assigned to the listed spoilage- or clinically associated taxa in reconstituted-culture kombucha, in contrast to their detection in back-slopping fermentation. Nevertheless, this observation does not establish microbial viability or complete exclusion, and further culture-based validation is warranted.

Overall, these findings demonstrate that rational microbial reconstitution can improve the quality and safety of kombucha. While the results provide a strong experimental foundation, broader implications such as industrial scalability should be considered future directions rather than established outcomes. Further work is needed to optimize strain ratios, verify reproducibility across independent large-scale trials, and evaluate process stability under industrial conditions.

One limitation of the present study is that we did not include an uninoculated sugared tea control. The primary aim of our work was to compare the fermentation performance and metabolite production among different reconstituted microbial consortia, rather than to distinguish between microbial and spontaneous chemical changes. While the increase in polyphenol content and related metabolites is consistent with previous findings on kombucha fermentation, the absence of an uninoculated control prevents us from fully ruling out non-microbial contributions. Future studies will incorporate uninoculated tea media as baseline controls, in order to better differentiate spontaneous chemical transformations from microbially driven changes.

## Data Availability

The data presented in the study are deposited in the figshare repository, accession link https://doi.org/10.6084/m9.figshare.30526916.
